# Supplementary material for: Enhancing Stability of Cu/ZnO Catalysts in the CO2 Hydrogenation to Methanol by the Addition of MoO3 and ReO3 Promoters
Source: Nanomaterials (Basel). 2025 Nov 17;15(22):1730. doi: 10.3390/nano15221730 (PMC12655111; doi:10.3390/nano15221730)
Supplement: Supplementary file 1 [file nanomaterials-15-01730-s001.zip › nanomaterials-3960613-supplementary.pdf]

SUPPORTING INFORMATION

Enhancing Stability of Cu/ZnO Catalysts in the CO_2 Hydrogenation to Methanol by the Addition of MoO_3 and ReO_3 Promoters

by

Jose Soriano Rodríguez [1], José Manuel López Nieto [1], Enrique Rodriguez-Castellón [2], Antonia Infantes [2], Daviel Gómez [1] and Patricia Concepción [1,*]

[1] Instituto de Tecnología Química, Universitat Politècnica de València-Consejo Superior de Investigaciones Científicas (UPV-CSIC), Avenida de los Naranjos s/n, 46022 Valencia, Spain

[2] Departamento de Química Inorgánica, Facultad de Ciencias, Cristalografía y Mineralogía, Instituto Interuniversitario de Investigación en Biorrefinerías I3B, Universidad de Málaga, Campus de Teatinos, 29071 Málaga, Spain

* To whom correspondence should be addressed.

Email address: pconcepc@itq.upv.es

Table of contents

Table S1. X-ray photoelectron spectroscopy (XPS) at the laboratory scale. BE (eV) of the $Cu2p_{3/2}$, $Zn2p_{3/2}$, $Re4f_{7/2}$, $Mo3d_{5/2}$ and O1s core levels and modified Auger parameter (α′=BE+KE) of the Cu $2p_{3/2}$ and Zn $2p_{3/2}$, quantitative surface composition and surface Cu/Zn and Cu/Me atomic ratio of reduced samples. 5

Table S2. Catalytic performance of all the catalysts [a] 6

Table S3. Catalytic Performance of all the catalysts. [a] 7

Table S4. Initial reaction rate of methanol and CO formation. 8

Table S5. Apparent energy for methanol and CO formation at 20 bar for all the catalysts. 10

Table S6. Thermogravimetric analysis of Cu/ZnO/Me-based catalysts. 11

Figure S1. XRD patterns of different CuZnMe-X-based catalysts: **A)** As-prepared; **B)** calcined at 360 °C; **C)** reduced at 200 °C; and, **D)** after reaction (i.e., with CO_2/H_2 molar ratio of 1/3, temperature in the 220-280 ºC temperature range, total reaction time of 8 h and a pressure of 20 bar). Catalysts: a) CuZn; b) CuZnRe-0.06; c) CuZnRe-0.50; d) CuZnRe-3.50; e) CuZnMo-0.06; f) CuZnMo-0.44 and g) CuZnMo-3.50. Symbols: (●) CuO (00-041-0254); (●) ZnO (01-079-0207); (●) Cu^0 (01-070-3038). 12

Figure S2. STEM-EDX microscopy studies after 80 h of reaction in CuZnMe-X catalysts: A) CuZn; B) CuZnRe-0.5 and C) CuZnMo-3.5........13

Figure S3. X-ray photoelectron spectroscopy (XPS) studies on CuZnMe-X-based catalysts: A) Na1s peak of samples promoted with rhenium reduced in H2 at 200 ºC and B) Na1s peak of samples promoted with molybdenum reduced in H2 at 200 ºC. Catalysts: (a) CuZn; (b) CuZnRe-0.06; (c) CuZnRe-0.50; (d) CuZnMo-0.44 and (e) CuZnMo-3.50........14

Figure S4. X-ray photoelectron spectroscopy (XPS) studies on CuZnMe-X-based catalysts: **A)** Cu2p peak of samples promoted with rhenium reduced in H_2 at 200 ºC; **B)** Cu2p peak of samples promoted with molybdenum reduced in H_2 at 200 ºC; **C)** Zn2p peak of samples promoted with rhenium reduced in H_2 at 200 ºC; and, **D)** Zn2p peak of samples promoted with molybdenum reduced in H_2 at 200 ºC. Catalysts: (-1-) CuZnRe-0.06; (-2-) CuZnRe-0.50; (-3-) CuZnRe-3.50; (-4-) CuZnMo-0.06; (-5-) CuZnMo-0.44 and (-6-) CuZnMo-3.50. 15

Figure S5. X-ray photoelectron spectroscopy (XPS) studies on CuZnMe-X-based catalysts: **A)** $CuL_3M_{45}M_{45}$-Auger peak of samples promoted with rhenium reduced in H_2 at 200 ºC; **B)** $CuL_3M_{45}M_{45}$-Auger peak of samples promoted with molybdenum reduced in H_2 at 200 ºC; **C)** Zn $L_3M_{45}M_{45}$-Auger peak of samples promoted with rhenium reduced in H_2 at 200 ºC; and, **D)** Zn $L_3M_{45}M_{45}$-Auger peak of samples promoted with molybdenum reduced in H_2 at 200 ºC. Catalysts:

(-1-) CuZnRe-0.06; (-2-) CuZnRe-0.50; (-3-) CuZnRe-3.50; (-4-) CuZnMo-0.06; (-5-) CuZnMo-0.44 and (-6-) CuZnMo-3.50. 16

Figure S6. X-ray photoelectron spectroscopy (XPS) studies on the un-promoted Cu/ZnO-based catalysts: **A)** Cu2p peak of un-promoted sample reduced in H_2 at 200 ºC; **B)** Cu $L_3M_{45}M_{45}$-Auger peak of un-promoted sample reduced in H_2 at 200 ºC; **C)** Zn2p peak of un-promoted sample reduced in H_2 at 200 ºC; and, **D)** Zn $L_3M_{45}M_{45}$-Auger peak of un-promoted sample reduced in H_2 at 200 ºC. 17

Figure S7. X-ray photoelectron spectroscopy (XPS) studies on CuZnMe-X-based catalysts: **A)** Re4f peak of samples promoted with rhenium reduced in H_2 at 200 ºC; and **B)** Mo3d peak of samples promoted with molybdenum reduced in H_2 at 200 ºC. Catalysts: (-2-) CuZnRe-0.50; (-3-) CuZnRe-3.50; (-4-) CuZnMo-0.06; (-5-) CuZnMo-0.44 and (-6-) CuZnMo-3.50. 18

Figure S8. Reference Raman spectra of copper-based species recorded at room temperature using a 785 nm diode laser. **A)** CuO and **B)** $Cu(OH)_2$. 19

Figure S9. In situ Raman spectroscopic studies on CuZnMe-X catalysts (recorded with 785 nm diode laser). **A)** Raman spectra of samples promoted with rhenium under reaction conditions, i.e., CO_2/H_2 (molar ratio 1/3) at 260 °C; and **B)** Raman spectra of samples promoted with molybdenum under reaction conditions, i.e., CO_2/H_2 (molar ratio 1/3) at 260 °C. Catalysts: (-1-) CuZnRe-0.06; (-2-) CuZnRe-0.50; (-3-) CuZnRe-3.50; (-4-) CuZnMo-0.06; (-5-) CuZnMo-0.44 and (-6-) CuZnMo-3.50. 20

Figure S10. In situ Raman spectroscopic studies on CuZnMo-3.50 catalyst (recorded with 785 nm diode laser). **A)** Comparative analysis of calcined and reduced sample; and **B)** comparative analysis of reduced and spent (i.e., after reaction) sample. 21

Figure S11. Temperature-programmed desorption of CO_2 (TPD-CO_2) studies on reduced CuZnMe-X-based catalysts. Catalysts: (-1-) CuZn; (-2-) CuZnRe-0.06; (-3-) CuZnRe-0.50; (-4-) CuZnRe-3.50; (-5-) CuZnMo-0.06; and (-6-) CuZnMo-0.44 22

Figure S12. Space–time yield (STY) of (A,B) methanol and (C,D) CO expressed per gram of catalyst as a function of Me loading for (A,C) CuZnRe-X and (B,D) CuZnMo-X catalysts at 20 bar. Reaction conditions: H_2/CO_2 = 3, 220-280 °C and a WHSV of 30000 mL/h g_{cat}. 23

Figure S13. Space–time yield (STY) of (A,B) methanol and (C,D) CO expressed per surface area as a function of Me loading for (A,C) CuZnRe-X and (B,D) CuZnMo-X catalysts at 20 bar. Reaction conditions: H_2/CO_2 = 3, 220-280 °C and a WHSV of 30000 mL/h g_{cat}. 24

Figure S14. Space–time yield of methanol (**A**) or CO (**B**) vs. contact time on Cu/ZnO/Me-based catalysts. Reaction conditions: 240 ºC, CO_2:H_2 1:3 molar ratio, 20 bar. Space–time yield in g/h m^2. 25

Figure S15. **(A,B)** Methanol initial rate per exposed Cu atom as a function of promoter loading for (A) CuZnRe-X and (B) CuZnMo-X catalysts. (**C,D**) Intrinsic CO initial rate per exposed Cu atom as a function of promoter loading for (C) CuZnRe-X and (D) CuZnMo-X catalysts, at 240

°C. Reaction conditions: H_2/CO_2 = 3 and 20 bar. The black dots represent the un-promoted CuZn sample. 26
Figure S16. Apparent activation energies (Eapp) for methanol and CO formation as a function of promoter loading for (A) CuZnRe-X and (B) CuZnMo-X catalysts. Reaction conditions: 20 bar, H_2/CO_2 = 3, 220-280 °C, WHSV = 30000 mL/h g_{cat}. 27
Figure S17. Long-term deactivation constants of CuZnMe-X catalysts. Reaction conditions: 240 ºC, CO_2:H_2 molar ratio of 1:3 and 20 bar. 28
Figure S18. Comparative catalytic performance of CuZnMe-X and CuZnOAl$_2$O$_3$ catalysts in the CO_2 hydrogenation at 20 bar and 240 ºC: A) Evolution of conversion of CO_2 with time on stream and B) Long-term deactivation constants of the catalysts.

Figure S19. Thermogravimetric analysis of CuZnRe-X-based catalysts, calcined in air at 360 ºC: **A)** CuZn (0.69%), **B)** CuZnRe-0.06 (0.83%), **C)** CuZnRe-0.50 (0.80%) and **D)** CuZnRe-3.50 (1.24%). The numerical values for each catalyst correspond to the percentage of mass loss with temperature. 30
Figure S20. Thermogravimetric analysis of CuZnMo-X-based catalysts**,** calcined in air at 360 ºC: **A)** CuZn (0.69%), **B)** CuZnMo-0.06 (1.15%), **C)** CuZnMo-0.44 (1.24%) and **D)** CuZnMo-3.50 (2.71%). The numerical values for each catalyst correspond to the percentage of mass loss with temperature. 31
Figure S21. Relationship between the deactivation rate constants obtained from catalytic time-on-stream studies and the weight loss determined by thermogravimetric (TG) analysis for the CuZnMe-X catalysts. 32
Figure S22. In situ Raman spectroscopic studies on CuZnMo-3.50 catalyst by co-adding ~3.1% H_2O/Ar (recorded with 785 nm diode laser). Conditions: (-1-) H_2 at 200 ºC, (-2-) Ar at 280 ºC, (-3-) ~3.1% H_2O/Ar at room temperature, and (-4-) ~3.1% H_2O/Ar at 200 ºC. 33

Table S1. X-ray photoelectron spectroscopy (XPS) at the laboratory scale. BE (eV) of the $Cu2p_{3/2}$, $Zn2p_{3/2}$, $Re4f_{7/2}$, $Mo3d_{5/2}$ and O1s core levels and modified Auger parameter (α′=BE+KE) of the Cu $2p_{3/2}$ and Zn $2p_{3/2}$, quantitative surface composition and surface Cu/Zn and Cu/Me atomic ratio of reduced samples.

Catalyst	Cu: O: Zn: Me	Ratio Cu/Zn	Ratio Cu/Me	Cu $2p_{3/2}$ BE, eV	α′	Zn $2p_{3/2}$ BE, eV	α′	Re $4f_{7/2}$ BE, eV	Mo $3d_{5/2}$ BE, eV	O 1s BE, eV
CuZn	24.4: 47.6: 28.0 :0.00	0.87	--	932.8	1851.4	1022.00	2010.00	--	--	
$CuZnRe_{0.06}$	18.9: 60.6: 20.4: 0.00	0.90	--	932.6	1851.0	1022.22	2010.00	--	--	530.8, 532.8
$CuZnRe_{0.50}$	11.5: 71.1: 17.0: 0.35	0.67	32.8	932.6	1850.9	1022.20	2010.00	43.3	--	530.6, 532.8
$CuZnRe_{3.50}$	14.8: 69.2: 15.4: 0.50	0.96	29.6	932.6	1851.0	1022.23	2009.90	44.2	--	530.6, 532.8
$CuZnMo_{0.06}$	8.7: 75.9: 11.1: 4.20	0.78	2.07	932.6	1850.8	1022.30	2010.15	--	233.3	530.8, 533.2
$CuZnMo_{0.44}$	19.5: 57.7: 18.9: 3.87	1.03	5.02	932.6	1851.0	1022.06	2010.07	--	229.8 (0.3%), 232.2 (3.5%)	530.7, 532.3
$CuZnMo_{3.50}$	10.4: 62.9: 13.6: 13.11	0.77	0.80	932.6	1851.1	1022.07	2010.24	--	230.8 (3.6%), 232.6 (9.5%)	530.8, 532.4

Table S2. Catalytic performance of all the catalysts [a]

Temperature (°C)	Catalyst	X_{CO2} (%)	Selectivity (%)		Space Time Yield MeOH and CO				
					STY_{MeOH}	STY_{CO}	STY_{MeOH}	STY_{CO}	Ratio STY_{MeOH}/ STY_{CO}
			S_{MeOH}	S_{CO}	(g/h g_{cat})		(g/h m^2)		
220	CuZn	4.1	91.3	8.3	0.35	0.027	0.034	0.003	11.3
	$CuZnRe_{0.06}$	4.8	90.0	9.6	0.40	0.038	0.044	0.004	11.0
	$CuZnRe_{0.50}$	4.1	90.7	8.8	0.35	0.029	0.041	0.003	13.7
	$CuZnRe_{3.50}$	3.5	91.1	8.3	0.30	0.024	0.034	0.003	11.3
	$CuZnMo_{0.06}$	5.8	86.2	13.4	0.47	0.063	0.034	0.005	6.8
	$CuZnMo_{0.44}$	5.0	86.8	12.8	0.41	0.053	0.014	0.002	7.0
	$CuZnMo_{3.50}$	1.4	90.2	8.4	0.12	0.009	0.005	0.0004	12.5
240	CuZn	5.5	83.8	15.9	0.43	0.071	0.042	0.007	6.0
	$CuZnRe_{0.06}$	6.2	83.0	16.7	0.48	0.084	0.052	0.009	5.8
	$CuZnRe_{0.50}$	5.6	84.3	15.3	0.43	0.069	0.051	0.008	6.4
	$CuZnRe_{3.50}$	4.2	85.0	14.6	0.33	0.050	0.039	0.006	6.5
	$CuZnMo_{0.06}$	7.7	74.5	25.3	0.54	0.160	0.039	0.012	3.3
	$CuZnMo_{0.44}$	8.1	74.9	24.9	0.59	0.165	0.023	0.005	4.6
	$CuZnMo_{3.50}$	2.1	83.6	15.5	0.16	0.027	0.007	0.001	7.0
260	CuZn	7.0	72.5	27.3	0.47	0.156	0.047	0.015	3.1
	$CuZnRe_{0.06}$	8.0	71.0	28.8	0.52	0.185	0.057	0.020	2.9
	$CuZnRe_{0.50}$	7.3	73.8	26.0	0.50	0.153	0.059	0.018	3.3
	$CuZnRe_{3.50}$	5.3	74.7	25.0	0.37	0.108	0.042	0.012	3.5
	$CuZnMo_{0.06}$	10.1	54.1	45.8	0.51	0.376	0.037	0.028	1.3
	$CuZnMo_{0.44}$	11.5	57.1	42.8	0.62	0.399	0.022	0.014	1.6
	$CuZnMo_{3.50}$	2.7	71.2	28.1	0.18	0.062	0.007	0.002	3.5
280	CuZn	8.8	55.3	44.6	0.45	0.32	0.045	0.032	1.4
	$CuZnRe_{0.06}$	10.4	54.8	45.1	0.53	0.38	0.057	0.041	1.4
	$CuZnRe_{0.50}$	9.5	60.5	39.4	0.53	0.30	0.063	0.036	1.8
	$CuZnRe_{3.50}$	6.3	59.9	39.8	0.35	0.21	0.041	0.024	1.7
	$CuZnMo_{0.06}$	15.3	42.3	57.6	0.63	0.69	0.046	0.051	0.9
	$CuZnMo_{0.44}$	14.1	32.3	67.7	0.43	0.78	0.019	0.027	0.7
	$CuZnMo_{3.50}$	3.2	52.4	47.0	0.16	0.12	0.006	0.005	1.2

[a] Reaction conditions: H_2/CO_2 = 3; 20 bar, 220-280 ºC; 30000 mL/h g_{cat} (100 mL/min; 0.2 g_{cat})

Table S3. Catalytic performance of all the catalysts. [a]

WHSV (mL/h g_{cat})	Catalyst [b]	X_{CO2} (%)	Selectivity (%)		Yield (%)		Space Time Yield MeOH and CO			
							STY_{MeOH}	STY_{CO}	STY_{MeOH}	STY_{CO}
			S_{MeOH}	S_{CO}	Y_{MeOH}	Y_{CO}	(g/h g_{cat})		(g/h m^2)	
30000.0	CuZn	5.3	83.8	15.9	4.48	0.85	0.40	0.066	0.039	0.0065
30000.0	$CuZnRe_{0.06}$	5.2	84.1	15.6	4.36	0.81	0.39	0.063	0.043	0.0069
30000.0	$CuZnRe_{0.50}$	4.2	86.0	13.6	3.62	0.57	0.33	0.045	0.039	0.0054
15000.0	$CuZnRe_{3.50}$	5.6	83.8	15.9	4.65	0.88	0.21	0.035	0.024	0.0041
30000.0	$CuZnMo_{0.06}$	7.0	80.3	19.5	5.65	1.37	0.51	0.109	0.038	0.0080
30000.0	$CuZnMo_{0.44}$	6.9	80.7	19.1	5.58	1.32	0.50	0.104	0.018	0.0037
13333.3	$CuZnMo_{3.50}$	5.9	77.0	22.4	4.56	1.33	0.18	0.046	0.007	0.0019
22222.2	CuZn	6.1	82.5	17.2	5.06	1.06	0.34	0.061	0.033	0.0061
22222.2	$CuZnRe_{0.06}$	5.4	83.4	16.3	4.48	0.87	0.30	0.051	0.033	0.0056
22222.2	$CuZnRe_{0.50}$	4.5	85.6	14.0	3.82	0.62	0.26	0.037	0.031	0.0044
11111.1	$CuZnRe_{3.50}$	5.6	83.3	16.3	4.65	0.91	0.16	0.027	0.018	0.0031
22222.2	$CuZnMo_{0.06}$	7.3	78.4	21.4	5.72	1.56	0.39	0.093	0.029	0.0068
22222.2	$CuZnMo_{0.44}$	7.7	78.4	21.4	6.05	1.65	0.41	0.098	0.014	0.0034
10000.0	$CuZnMo_{3.50}$	6.8	74.9	24.6	5.08	1.67	0.15	0.044	0.006	0.0017
15000.0	CuZn	7.3	79.6	20.1	5.83	1.47	0.26	0.057	0.025	0.0056
15000.0	$CuZnRe_{0.06}$	6.2	81.1	18.6	5.06	1.16	0.23	0.045	0.025	0.0049
15000.0	$CuZnRe_{0.50}$	5.3	83.8	15.9	4.41	0.84	0.20	0.033	0.024	0.0039
7500.0	$CuZnRe_{3.50}$	6.3	81.0	18.7	5.11	1.18	0.12	0.023	0.013	0.0027
15000.0	$CuZnMo_{0.06}$	8.2	74.5	25.3	6.13	2.09	0.28	0.083	0.020	0.0061
15000.0	$CuZnMo_{0.44}$	9.2	75.5	24.3	6.93	2.24	0.31	0.088	0.011	0.0031
6593.4	$CuZnMo_{3.50}$	8.2	71.5	28.1	5.86	2.31	0.12	0.040	0.005	0.0016
7500.0	CuZn	9.4	73.3	26.5	6.92	2.50	0.15	0.048	0.015	0.0048
7500.0	$CuZnRe_{0.06}$	8.1	75.1	24.7	6.06	2.00	0.14	0.039	0.015	0.0043
7500.0	$CuZnRe_{0.50}$	6.9	79.0	20.7	5.48	1.44	0.12	0.028	0.015	0.0025
3726.7	$CuZnRe_{3.50}$	8.0	75.5	24.3	6.01	1.93	0.07	0.019	0.008	0.0022
7500.0	$CuZnMo_{0.06}$	10.2	65.9	33.9	6.74	3.47	0.15	0.069	0.011	0.0050
7500.0	$CuZnMo_{0.44}$	11.7	68.4	31.4	8.02	3.68	0.18	0.073	0.006	0.0026
3314.9	$CuZnMo_{3.50}$	10.8	63.8	35.7	6.89	3.86	0.07	0.034	0.003	0.0013

[a] Reaction conditions: H_2/CO_2 = 3; 20 bar, 240 °C, 30000-3333 mL h^{-1} g_{cat}^{-1} (100-25 mL min^{-1}; 0.2-0.45 g_{cat})

Table S4. Initial reaction rate of methanol and CO formation.

Catalyst [a]	r_{MeOH} ($mol_{MeOH}/h\ mol_{Cu\text{-}surface}$)	r_{CO} ($mol_{CO}/h\ mol_{Cu\text{-}surface}$)	r_{MeOH} ($g_{MeOH}/h\ m^2$)	r_{CO} ($g_{CO}/h\ m^2$)	*Ratio* r_{MeOH}/r_{CO}
CuZn	35.1	3.6	0.112	0.010	10.9
$CuZnRe_{0.06}$	39.1	5.7	0.237	0.030	8.0
$CuZnRe_{0.50}$	17.3	2.2	0.168	0.019	8.9
$CuZnRe_{3.50}$	22.3	2.9	0.097	0.011	9.0
$CuZnMo_{0.06}$	41.4	5.3	0.162	0.018	9.0
$CuZnMo_{0.44}$	28.8	3.7	0.054	0.006	9.2
$CuZnMo_{3.50}$	7.8	1.5	0.018	0.003	6.9

[a] Reaction conditions: 240 ºC, CO_2:H_2 molar ratio of 1:3, and 20 bar.

Regarding the MeOH/CO ratio, it is important to note that this parameter merely reflects the competition between two parallel surface reactions occurring on a common site and by itself it does not provide direct information on the electronic or structural nature of the active sites, according to the general Langmuir–Hinshelwood (L-H) expression for two products formed on the same site ***x***.

$$r_{MeOH} = \frac{k_{MeOH} \prod_j (K_j P_j)^{v_{j,MeOH}}}{(1 + Species\ adsorbed\ on\ site\ x)^n} \quad ; \quad r_{CO} = \frac{k_{CO} \prod_j (K_j P_j)^{v_{j,CO}}}{(1 + Species\ adsorbed\ on\ site\ x)^n}$$

where r is the reaction rate, k is the kinetic constant of the reaction, K_j is the adsorption constants of species j, P_j is the partial pressures of reactants j, ν_j is the partial order of reactant j, and n is the total number of species competing for active sites.

Thus, the ratio between the methanol and CO formation rates can be written as follows:

$$\frac{r_{MeOH}}{r_{CO}} = \frac{\dfrac{k_{MeOH} \prod_j (K_j P_j)^{v_{j,MeOH}}}{(1 + Species\ adsorbed\ on\ site\ x)^n}}{\dfrac{k_{CO} \prod_j (K_j P_j)^{v_{j,CO}}}{(1 + Species\ adsorbed\ on\ site\ x)^n}} = \frac{k_{MeOH} \prod_j (K_j P_j)^{v_{j,MeOH}}}{k_{CO} \prod_j (K_j P_j)^{v_{j,CO}}}$$

Since the adsorption constants K_j cancel out if they are similar for both routes, the dominant term is a function of the partial pressures and the differences in partial order. In the CO_2 hydrogenation, the relevant reactants are CO_2 and H_2. Therefore

$$\frac{r_{MeOH}}{r_{CO}} = \frac{k_{MeOH}}{k_{CO}} \left(\frac{P_{H_2}}{P_{CO_2}} \right)^{\Delta v}$$

where $\Delta \nu$ represents the difference in partial reaction orders between the two pathways.

This expression demonstrates that the MeOH/CO ratio is a dimensionless kinetic ratio governed by the relative rate constants and reaction orders, and it does not intrinsically quantify the reactivity or electronic nature of individual surface sites. Therefore, the constancy of the MeOH/CO ratio among most catalysts simply implies that both products share a common type of surface site, not that all catalysts possess identical active sites.

Table S5. Apparent energy for methanol and CO formation at 20 bar for all the catalysts.

Catalyst [a]	*Ea* (CH_3OH), kJ/mol	*Ea* (CO), kJ/mol
CuZn	22.6	92.4
$CuZnRe_{0.06}$	14.6	87.6
$CuZnRe_{0.50}$	16.3	88.6
$CuZnRe_{3.50}$	12.2	82.8
$CuZnMo_{0.06}$	14.8	92.3
$CuZnMo_{0.44}$	22.3	101.8
$CuZnMo_{3.50}$	36.8	97.4

[a] Reaction conditions: 220-280 ºC, CO_2:H_2 molar ratio of 1:3, and 20 bar.

Table S6. Thermogravimetric analysis of Cu/ZnO/Me-based catalysts.

Catalyst [a]	Weight, % Low temperature range, ~150 °C	Weight, % Total temperature range
CuZn	0.34	0.69
$CuZnRe_{0.06}$	0.33	0.83
$CuZnRe_{0.50}$	0.19	0.80
$CuZnRe_{3.50}$	0.53	1.24
$CuZnMo_{0.06}$	0.45	1.15
$CuZnMo_{0.44}$	1.24	1.24
$CuZnMo_{3.50}$	0.69	2.71

[a] Samples calcined in air at 360 ºC.

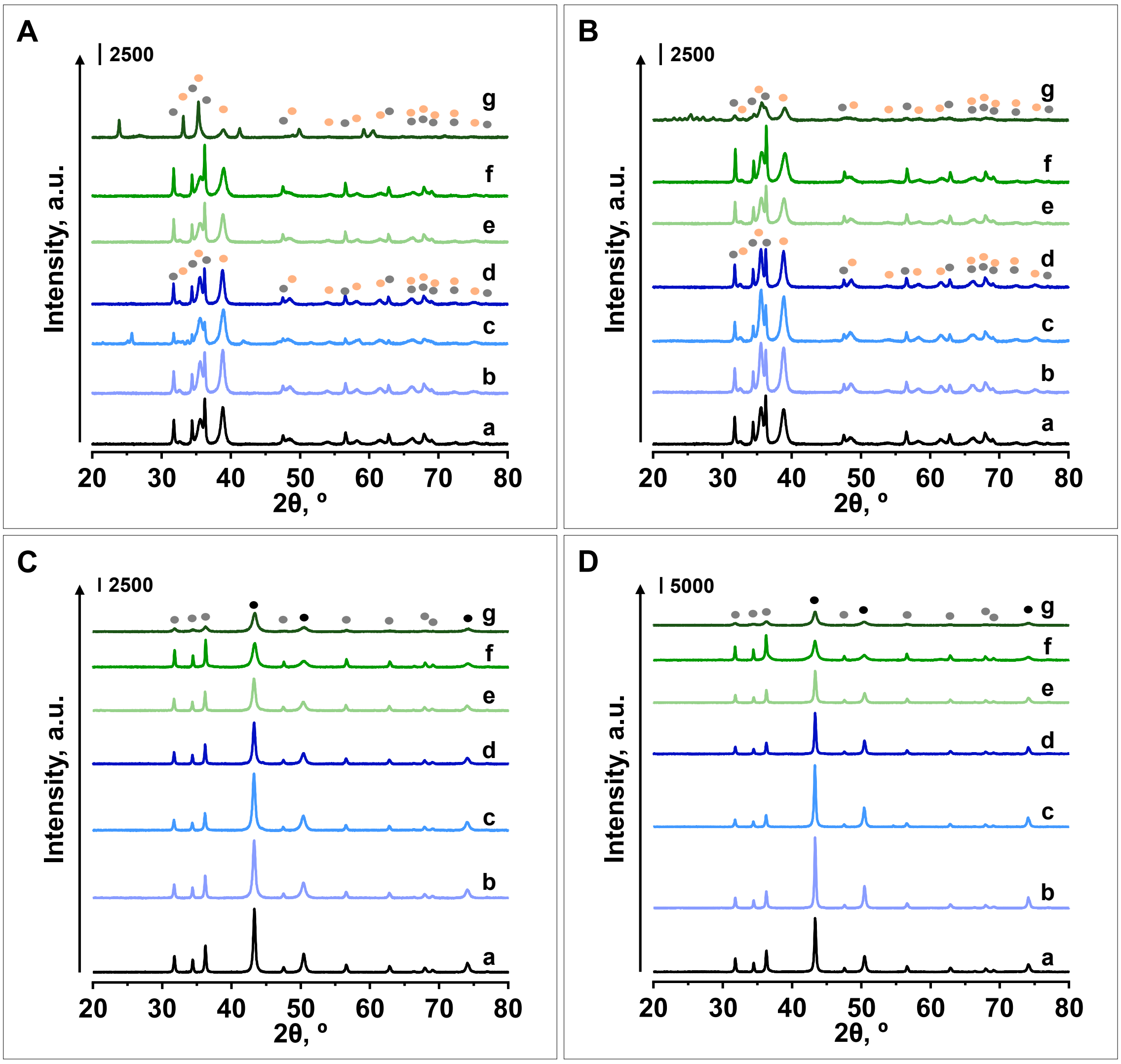

Figure S1. XRD patterns of different CuZnMe-X -based catalysts: **A)** As-prepared; **B)** calcined at 360 °C; **C)** reduced at 200 °C; and **D)** after reaction (i.e., with CO_2/H_2 molar ratio of 1/3, temperature in the 220-280 ºC temperature range, total reaction time of 8 h and a pressure of 20 bar). Catalysts: a) CuZn; b) CuZnRe-0.06; c) CuZnRe-0.50; d) CuZnRe-3.50; e) CuZnMo-0.06; f) CuZnMo-0.44 and g) CuZnMo-3.50. Symbols: (●) CuO (00-041-0254); (●) ZnO (01-079-0207); (●) Cu^0 (01-070-3038).

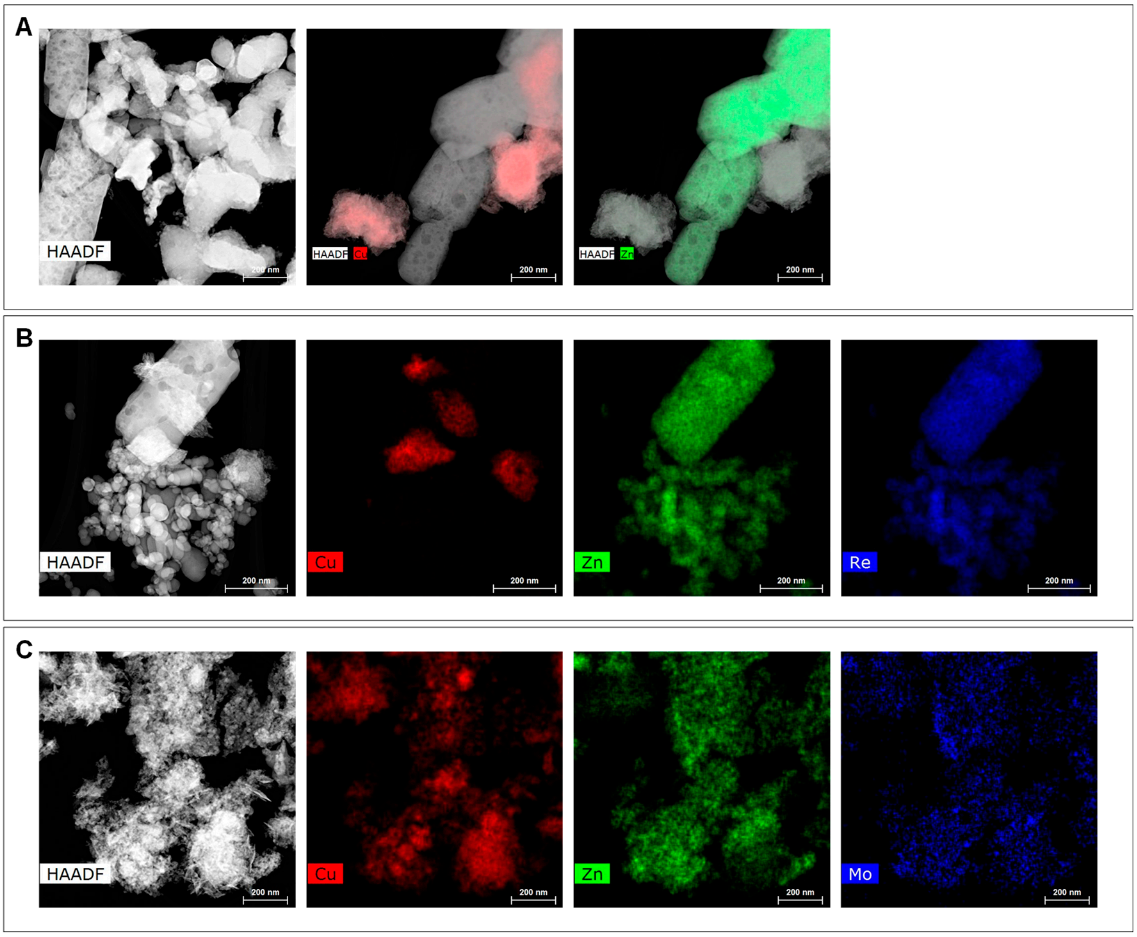

Figure S2. STEM-EDX microscopy studies after 80 h of reaction in CuZnMe-X catalysts: A) CuZn; B) CuZnRe-0.5 and C) CuZnMo-3.5.

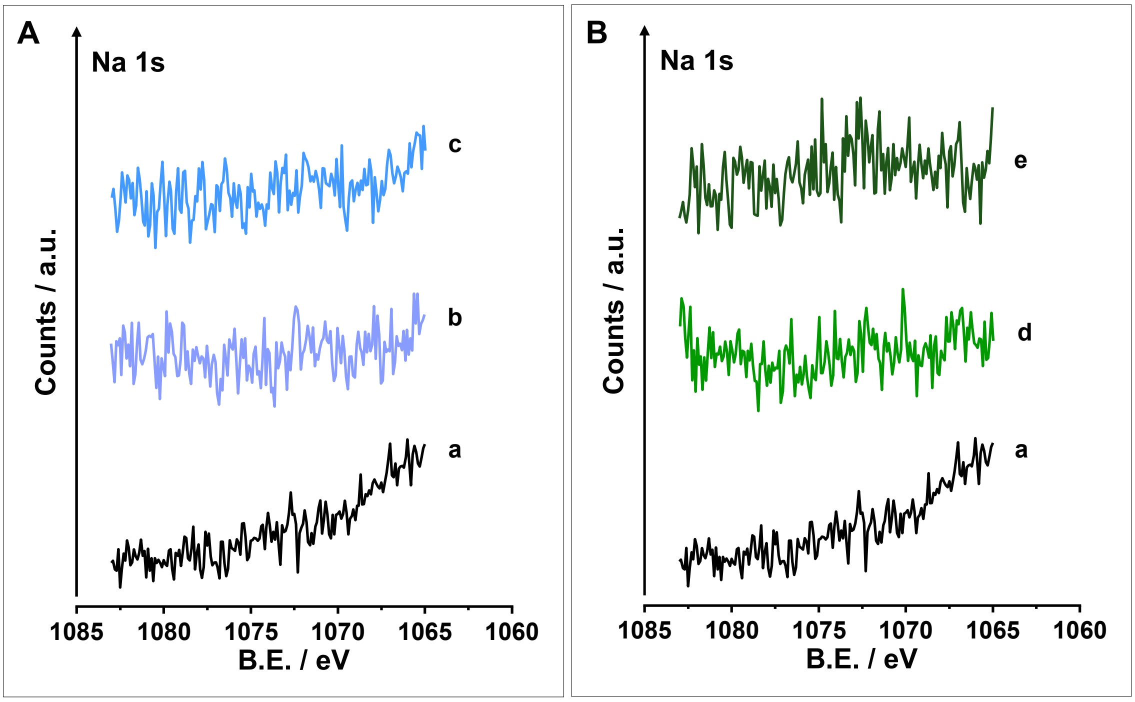

Figure S3. X-ray photoelectron spectroscopy (XPS) studies on CuZnMe-X-based catalysts: A) Na1s peak of samples promoted with rhenium reduced in H_2 at 200 ºC and B) Na1s peak of samples promoted with molybdenum reduced in H_2 at 200 ºC. Catalysts: (a) CuZn; (b) CuZnRe-0.06; (c) CuZnRe-0.50; (d) CuZnMo-0.44 and (e) CuZnMo-3.50.

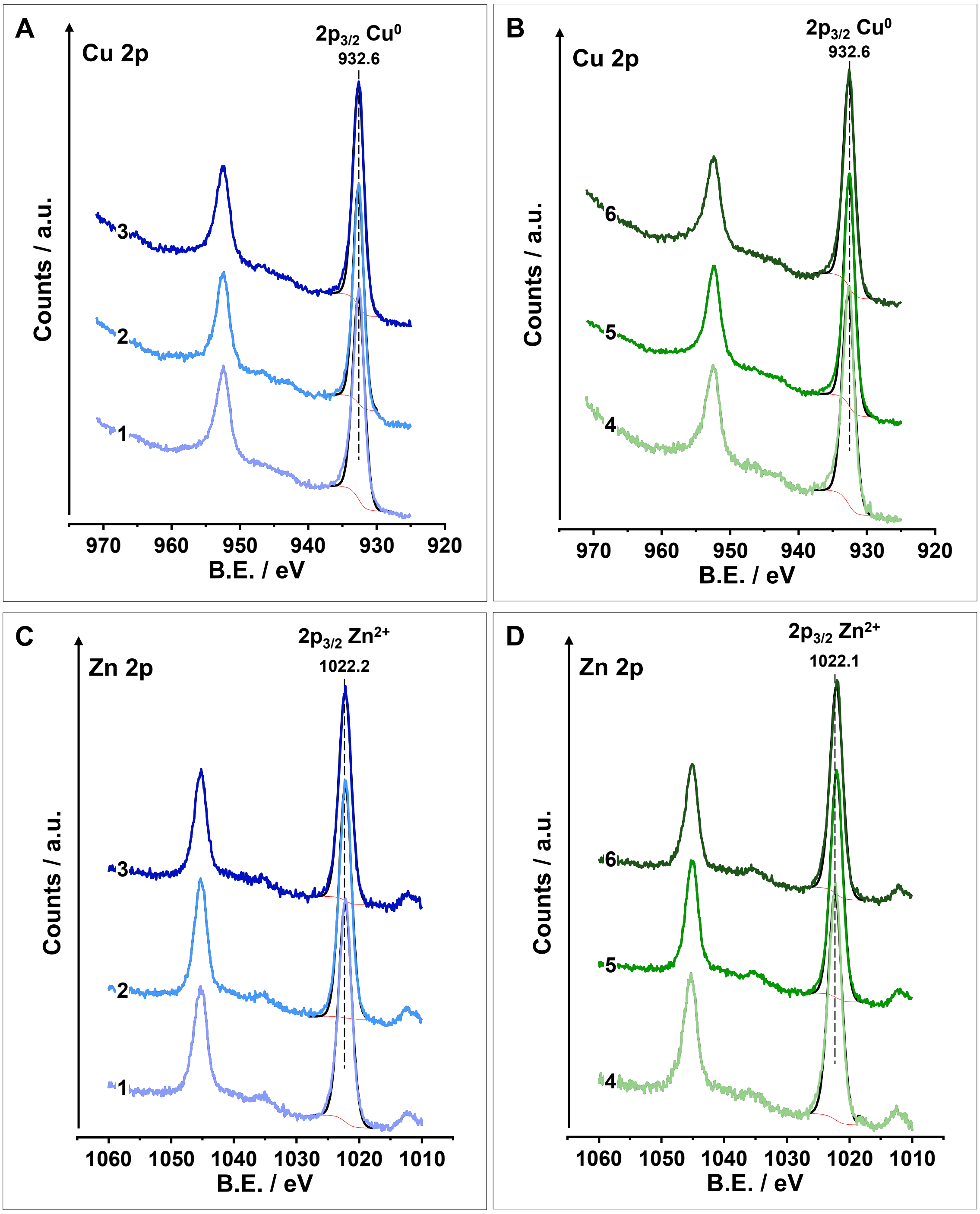

Figure S4. X-ray photoelectron spectroscopy (XPS) studies on CuZnMe-X-based catalysts: **A)** Cu2p peak of samples promoted with rhenium reduced in H_2 at 200 ºC; **B)** Cu2p peak of samples promoted with molybdenum reduced in H_2 at 200 ºC; **C)** Zn2p peak of samples promoted with rhenium reduced in H_2 at 200 ºC; and **D)** Zn2p peak of samples promoted with molybdenum reduced in H_2 at 200 ºC. Catalysts: (-1-) CuZnRe-0.06; (-2-) CuZnRe-0.50; (-3-) CuZnRe-3.50; (-4-) CuZnMo-0.06; (-5-) CuZnMo-0.44 and (-6-) CuZnMo-3.50.

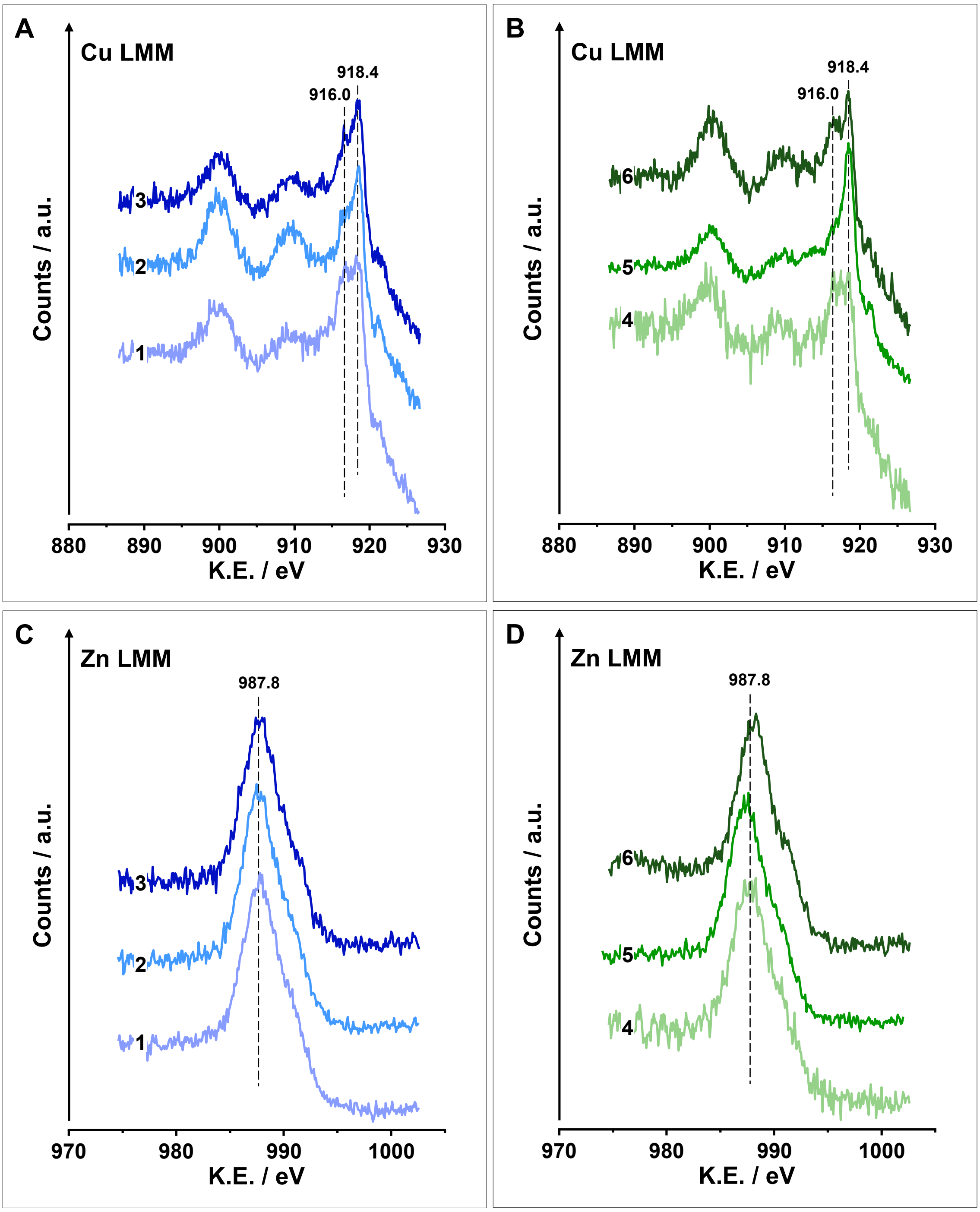

Figure S5. X-ray photoelectron spectroscopy (XPS) studies on CuZnMe-X-based catalysts: **A)** $CuL_3M_{45}M_{45}$-Auger peak of samples promoted with rhenium reduced in H_2 at 200 ºC; **B)** $CuL_3M_{45}M_{45}$-Auger peak of samples promoted with molybdenum reduced in H_2 at 200 ºC; **C)** Zn $L_3M_{45}M_{45}$-Auger peak of samples promoted with rhenium reduced in H_2 at 200 ºC; and **D)** Zn $L_3M_{45}M_{45}$-Auger peak of samples promoted with molybdenum reduced in H_2 at 200 ºC. Catalysts: (-1-) CuZnRe-0.06; (-2-) CuZnRe-0.50; (-3-) CuZnRe-3.50; (-4-) CuZnMo-0.06; (-5-) CuZnMo-0.44 and (-6-) CuZnMo-3.50.

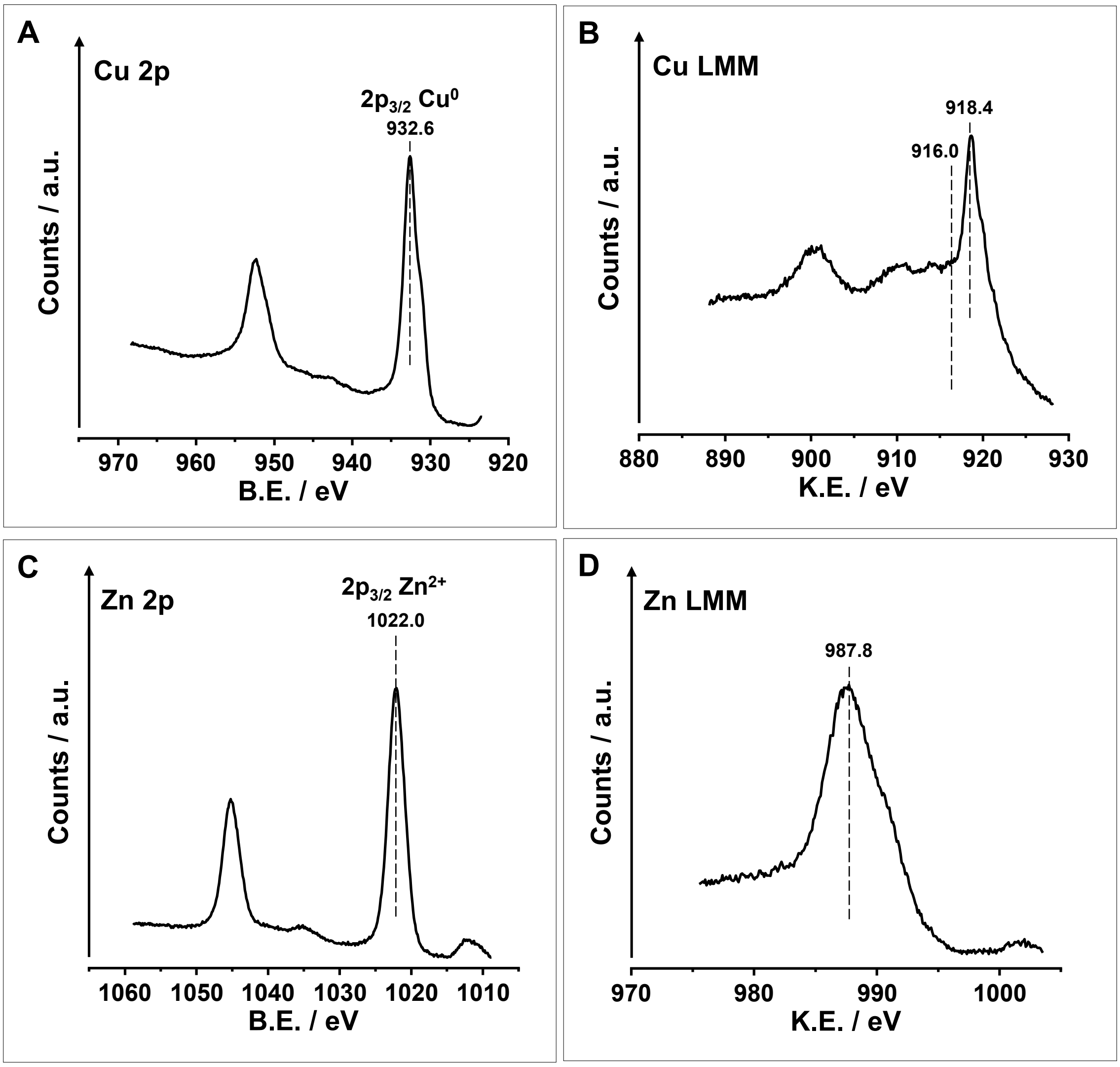

Figure S6. X-ray photoelectron spectroscopy (XPS) studies on the un-promoted Cu/ZnO-based catalysts: **A)** Cu2p peak of un-promoted sample reduced in H_2 at 200 ºC; **B)** Cu $L_3M_{45}M_{45}$-Auger peak of un-promoted sample reduced in H_2 at 200 ºC; **C)** Zn2p peak of un-promoted sample reduced in H_2 at 200 ºC; and **D)** Zn $L_3M_{45}M_{45}$-Auger peak of un-promoted sample reduced in H_2 at 200 ºC.

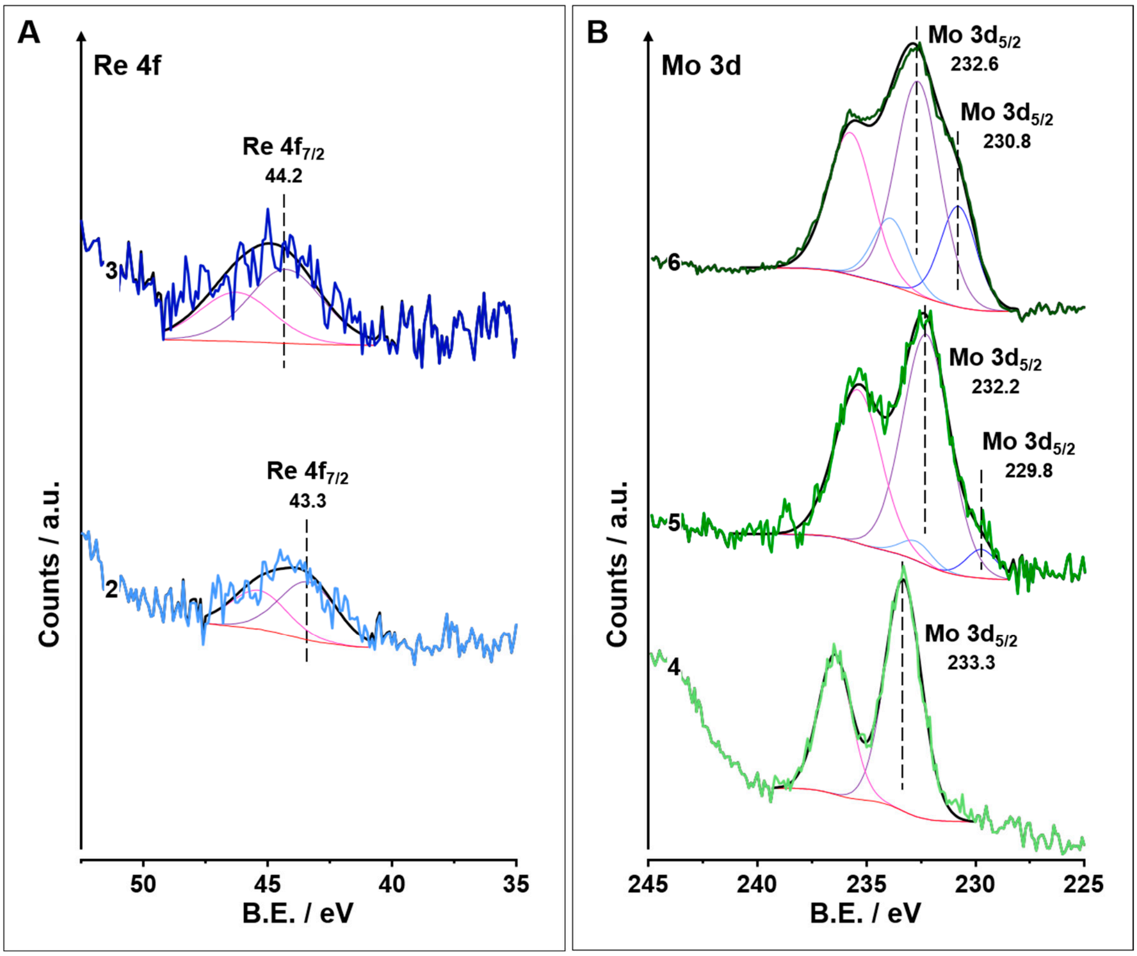

Figure S7. X-ray photoelectron spectroscopy (XPS) studies on CuZnMe-X-based catalysts: **A)** Re4f peak of samples promoted with rhenium reduced in H_2 at 200 ºC; and **B)** Mo3d peak of samples promoted with molybdenum reduced in H_2 at 200 ºC. Catalysts: (-2-) CuZnRe-0.50; (-3-) CuZnRe-3.50; (-4-) CuZnMo-0.06; (-5-) CuZnMo-0.44 and (-6-) CuZnMo-3.50.

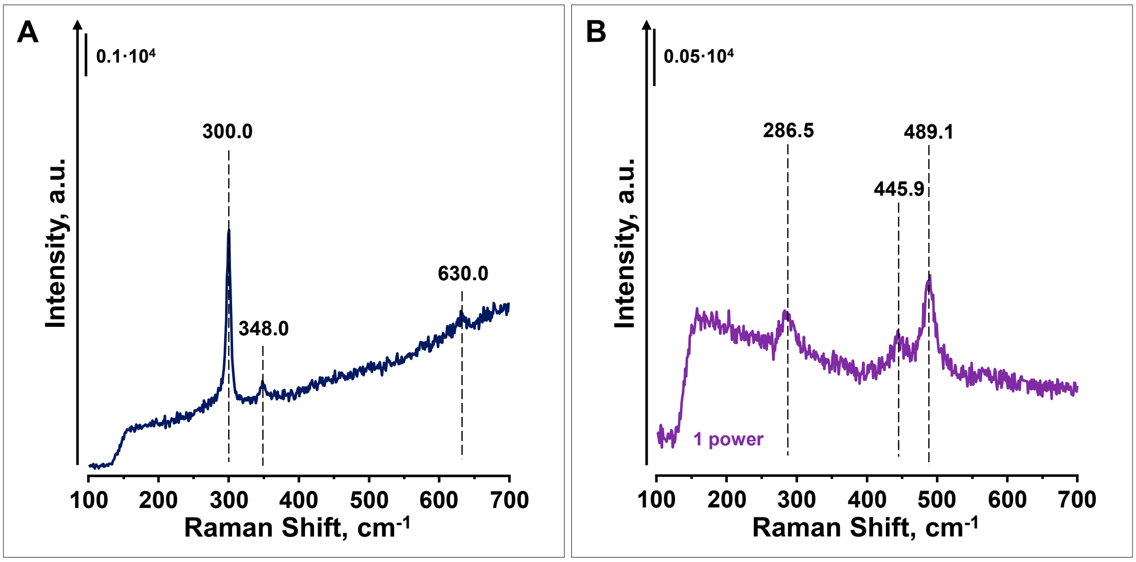

Figure S8. Reference Raman spectra of copper-based species recorded at room temperature using a 785 nm diode laser. **A)** CuO and **B)** $Cu(OH)_2$.

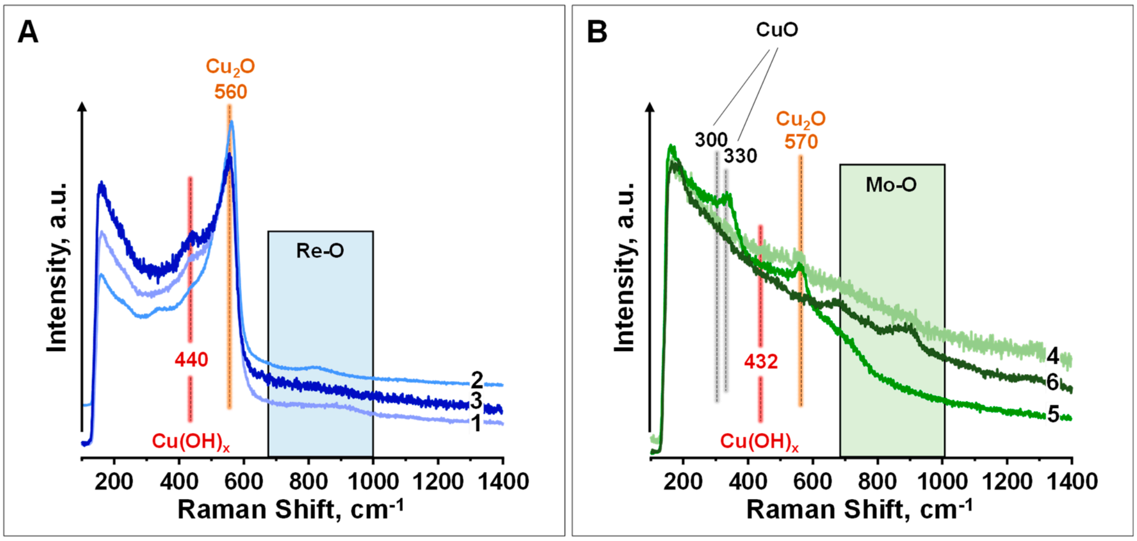

Figure S9. In situ Raman spectroscopic studies on CuZnMe-X catalysts (recorded with 785 nm diode laser). **A)** Raman spectra of samples promoted with rhenium under reaction conditions, i.e., CO_2/H_2 (molar ratio 1/3) at 260 °C; and **B)** Raman spectra of samples promoted with molybdenum under reaction conditions, i.e., CO_2/H_2 (molar ratio 1/3) at 260 °C. Catalysts: (-1-) CuZnRe-0.06; (-2-) CuZnRe-0.50; (-3-) CuZnRe-3.50; (-4-) CuZnMo-0.06; (-5-) CuZnMo-0.44 and (-6-) CuZnMo-3.50.

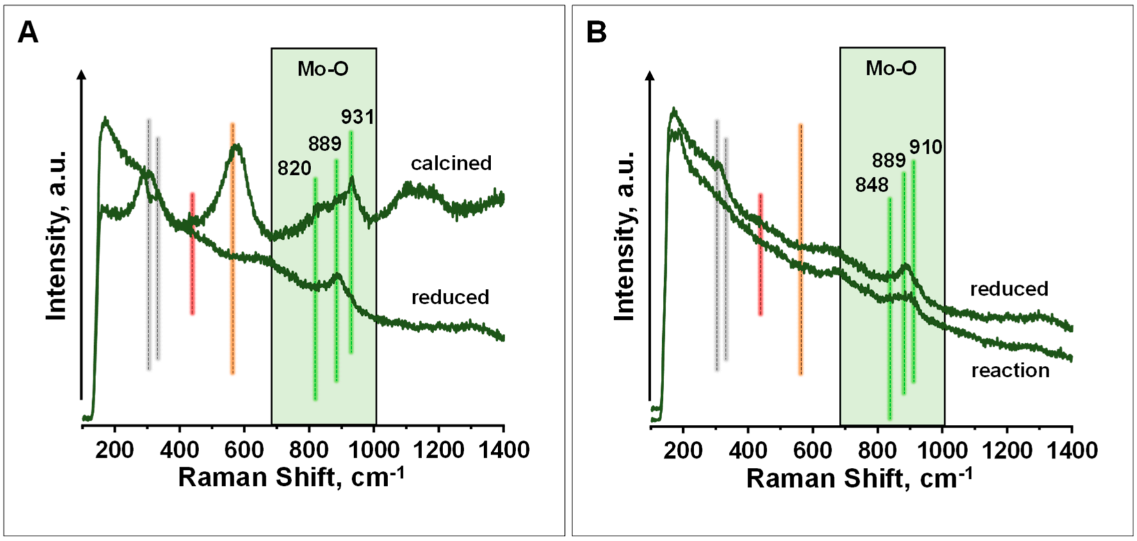

Figure S10. In situ Raman spectroscopic studies on CuZnMo-3.50 catalyst (recorded with 785 nm diode laser). **A)** Comparative analysis of calcined and reduced sample; and **B)** comparative analysis of reduced and spent (i.e., after reaction) sample.

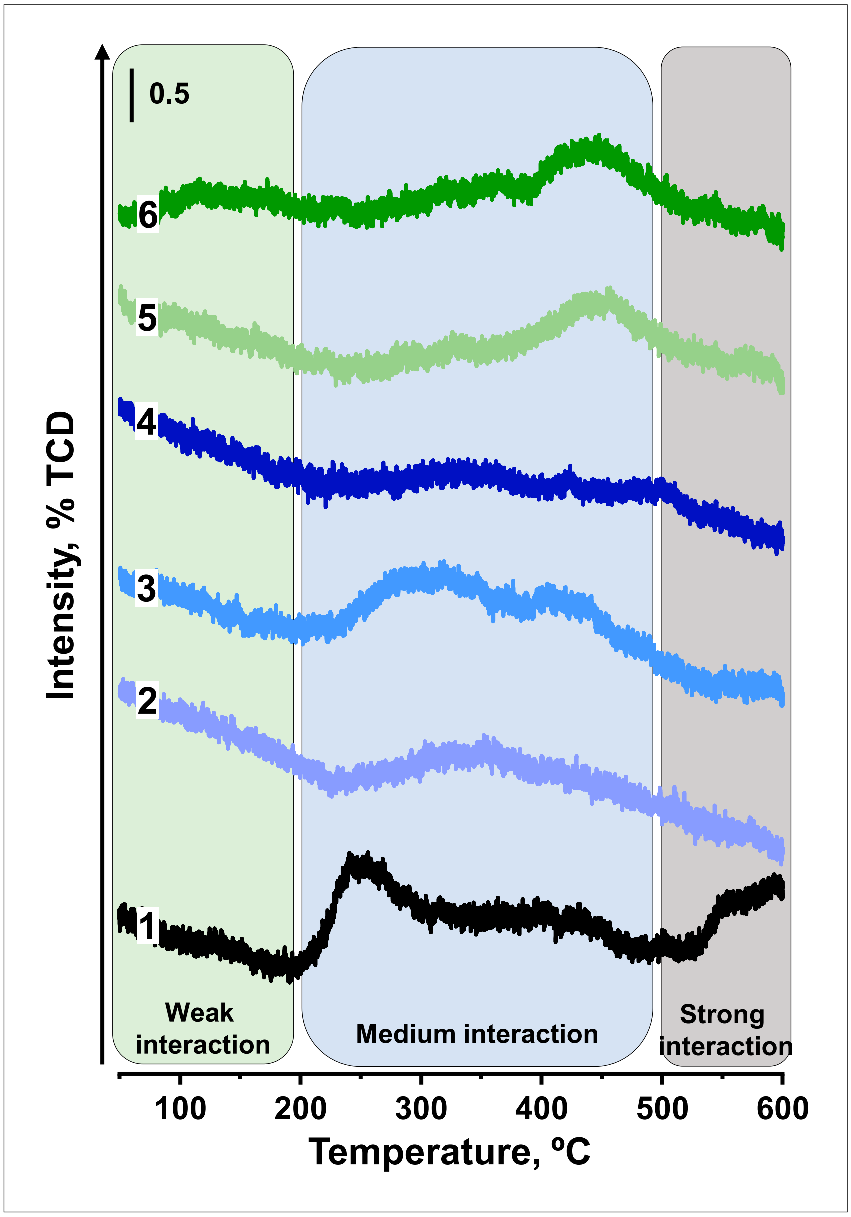

Figure S11. Temperature-programmed desorption of CO_2 (TPD-CO_2) studies on reduced CuZnMe-X-based catalysts. Catalysts: (-1-) CuZn; (-2-) CuZnRe-0.06; (-3-) CuZnRe-0.50; (-4-) CuZnRe-3.50; (-5-) CuZnMo-0.06; and (-6-) CuZnMo-0.44.

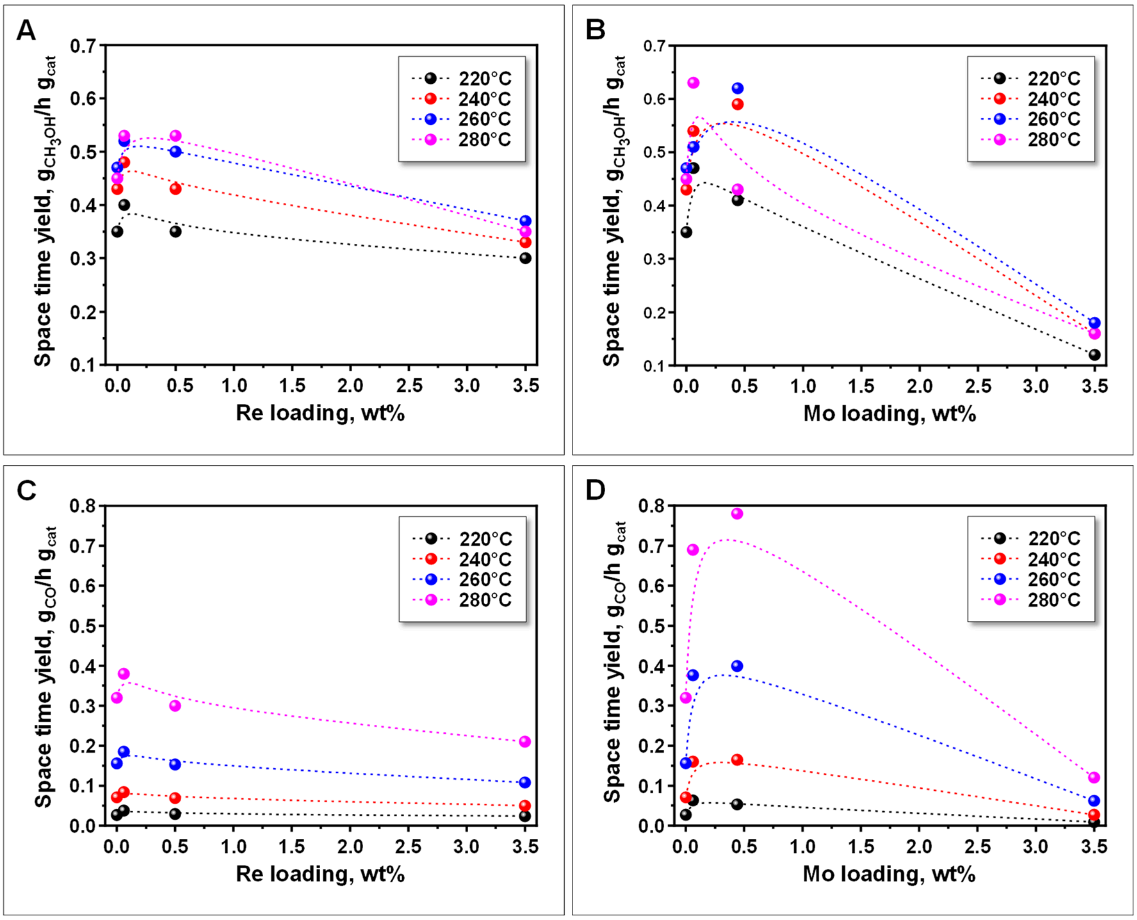

Figure S12. Space–time yield (STY) of (A,B) methanol and (C,D) CO expressed per gram of catalyst as a function of Me loading for (A,C) CuZnRe-X and (B,D) CuZnMo-X catalysts at 20 bar. Reaction conditions: H_2/CO_2 = 3, 220-280 °C and a WHSV of 30000 mL/h g_{cat}.

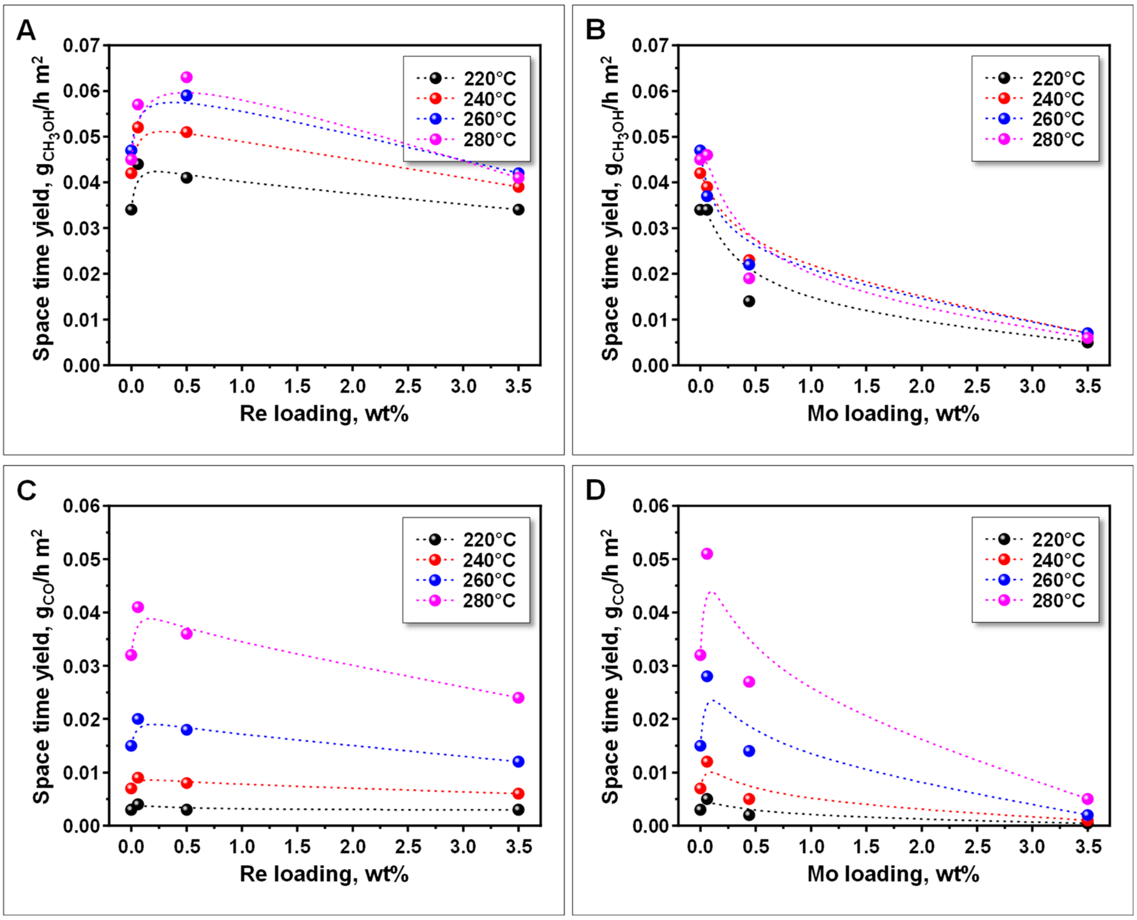

Figure S13. Space–time yield (STY) of (A,B) methanol and (C,D) CO expressed per surface area as a function of Me loading for (A,C) CuZnRe-X and (B,D) CuZnMo-X catalysts at 20 bar. Reaction conditions: $H_2/CO_2 = 3$, 220-280 °C and a WHSV of 30000 mL/h g_{cat}.

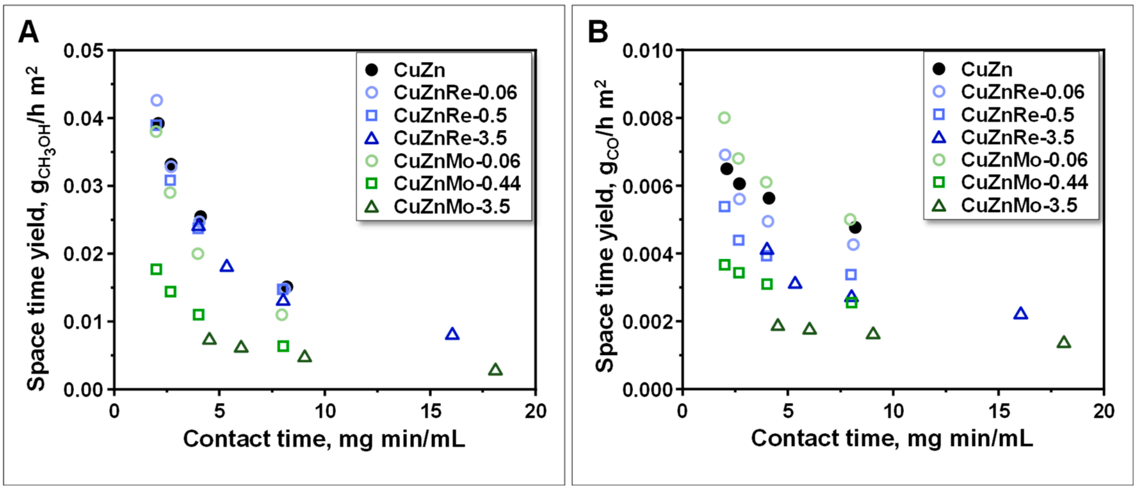

Figure S14. Space–time yield of methanol (**A**) or CO (**B**) vs. contact time on CuZnMe-X-based catalysts. Reaction conditions: 240 ºC, CO_2:H_2 1:3 molar ratio, 20 bar. Space–time yield in g/h m^2.

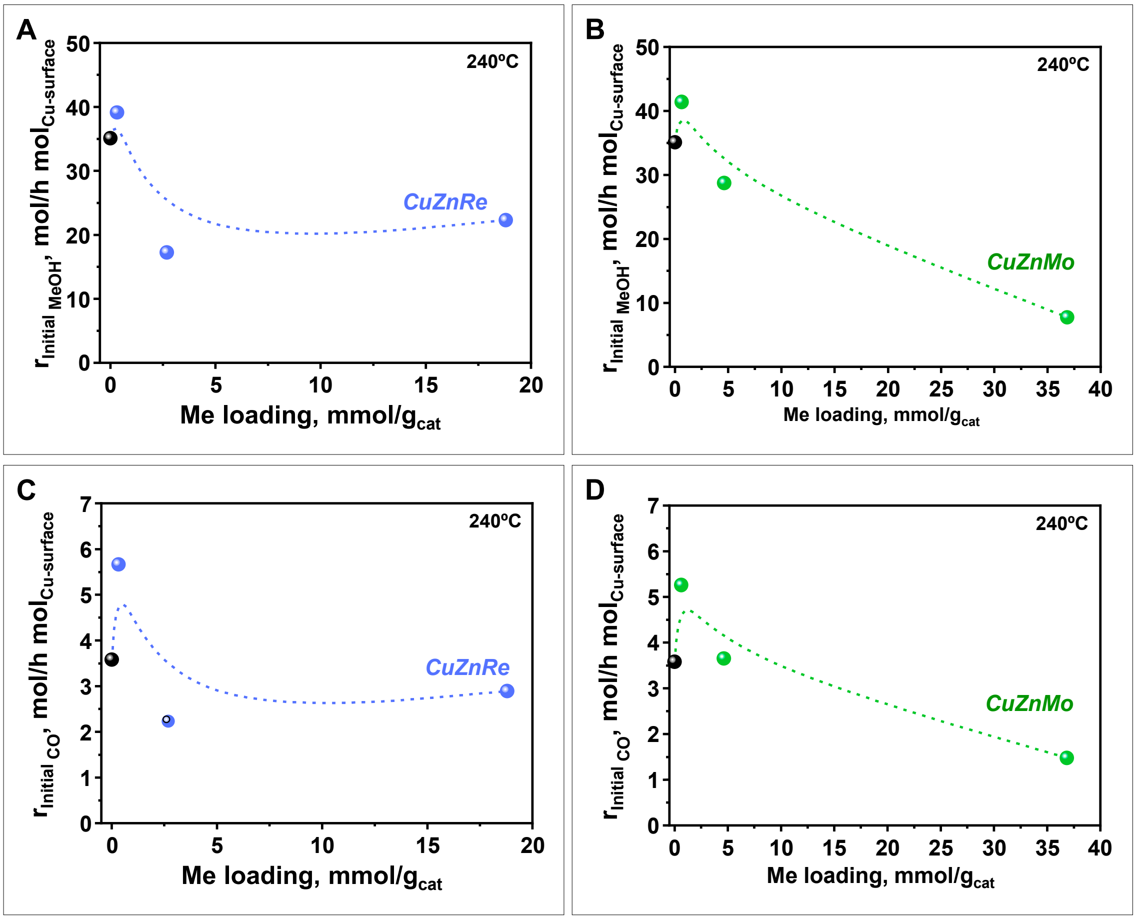

Figure S15. (A,B) Methanol initial rate per exposed Cu atom as a function of promoter loading for (A) CuZnRe-X and (B) CuZnMo-X catalysts. (**C,D**) Intrinsic CO initial rate per exposed Cu atom as a function of promoter loading for (C) CuZnRe-X and (D) CuZnMo-X catalysts, at 240 °C. Reaction conditions: H_2/CO_2 = 3 and 20 bar. The black dots represent the un-promoted CuZn sample.

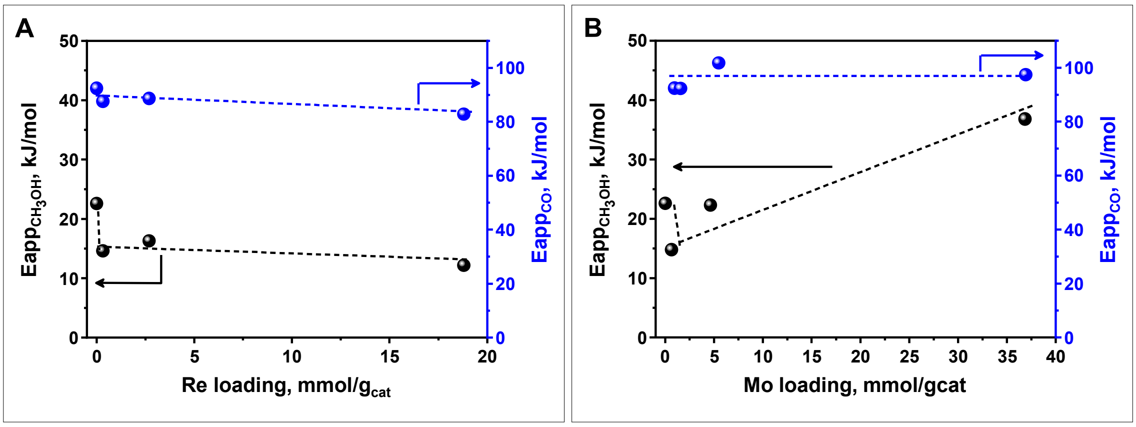

Figure S16. Apparent activation energies (Eapp) for methanol and CO formation as a function of promoter loading for (A) CuZnRe-X and (B) CuZnMo-X catalysts. Reaction conditions: 20 bar, $H_2/CO_2 = 3$, 220-280 °C, WHSV = 30000 mL/h g_{cat}.

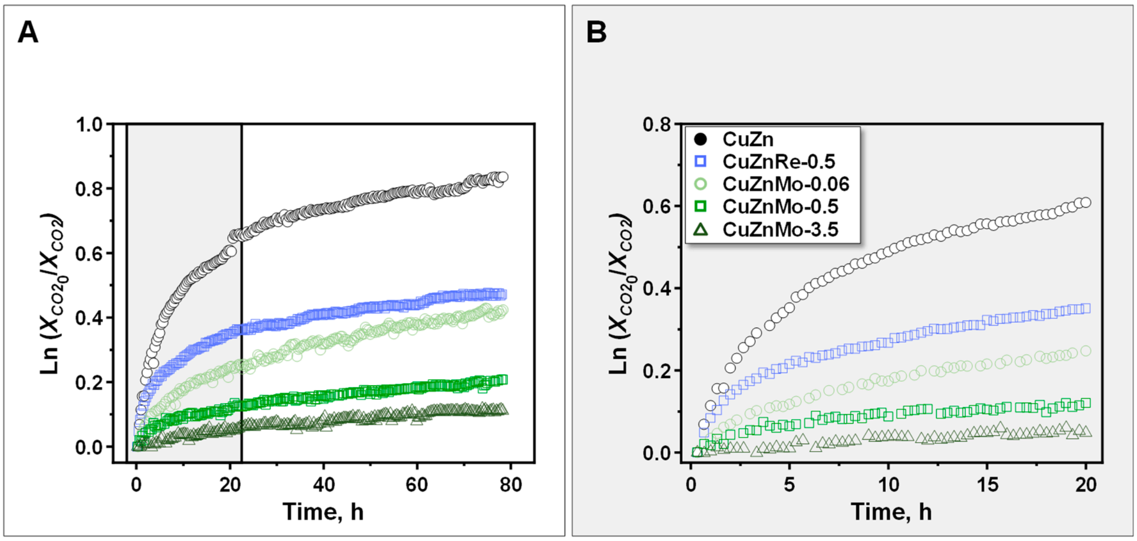

Figure S17. Long-term deactivation constants of CuZnMe-X catalysts. Reaction conditions: 240 ºC, CO_2:H_2 molar ratio of 1:3 and 20 bar.

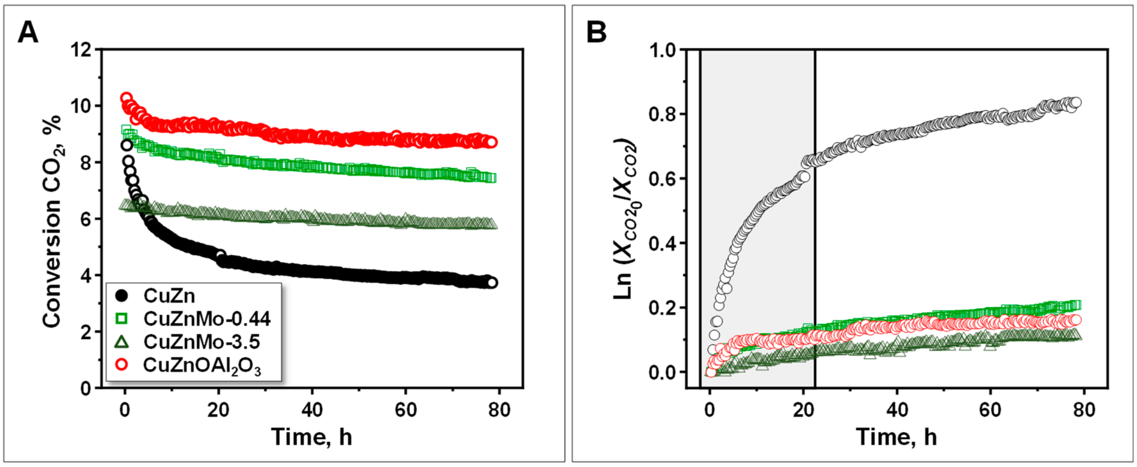

Figure S18. Comparative catalytic performance of CuZnMe-X and $CuZnOAl_2O_3$ catalysts in the CO_2 hydrogenation at 20 bar and 240 ºC: A) Evolution of conversion of CO_2 with time on stream and B) long-term deactivation constants of the catalysts.

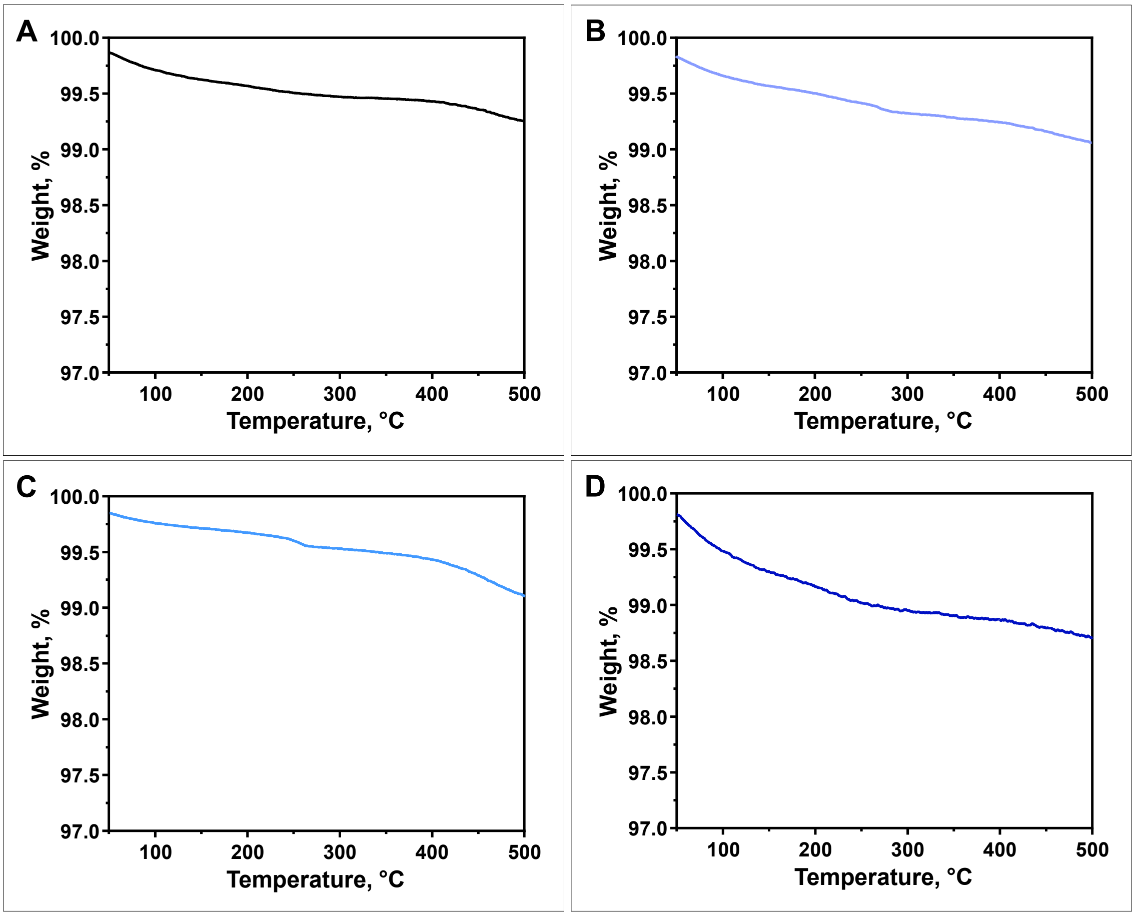

Figure S19. Thermogravimetric analysis of CuZnRe-X-based catalysts, calcined in air at 360 ºC: **A)** CuZn (0.69%), **B)** CuZnRe-0.06 (0.83%), **C)** CuZnRe-0.50 (0.80%), and **D)** CuZnRe-3.50 (1.24%). The numerical values for each catalyst correspond to the percentage of mass loss with temperature.

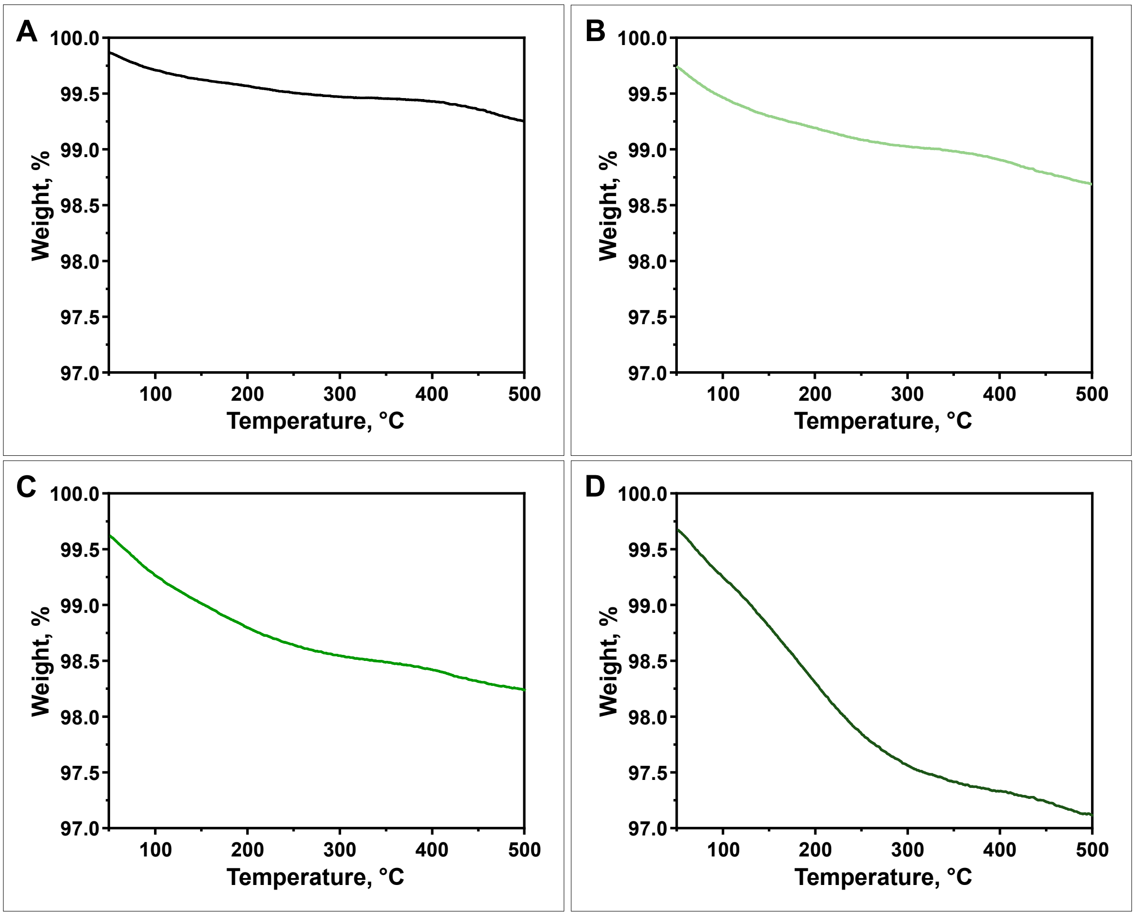

Figure S20. Thermogravimetric analysis of CuZnMo-X-based catalysts**,** calcined in air at 360 ºC: **A)** CuZn (0.69%), **B)** CuZnMo-0.06 (1.15%), **C)** CuZnMo-0.44 (1.24%), and **D)** CuZnMo-3.50 (2.71%). The numerical values for each catalyst correspond to the percentage of mass loss with temperature.

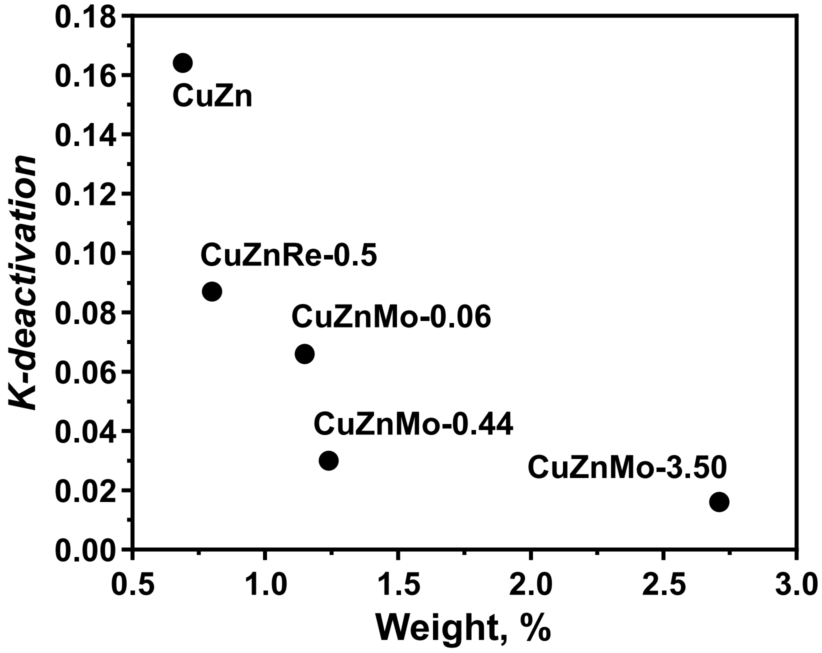

Figure S21. Relationship between the deactivation rate constants obtained from catalytic time-on-stream studies and the weight loss determined by thermogravimetric (TG) analysis for the CuZnMe-X catalysts.

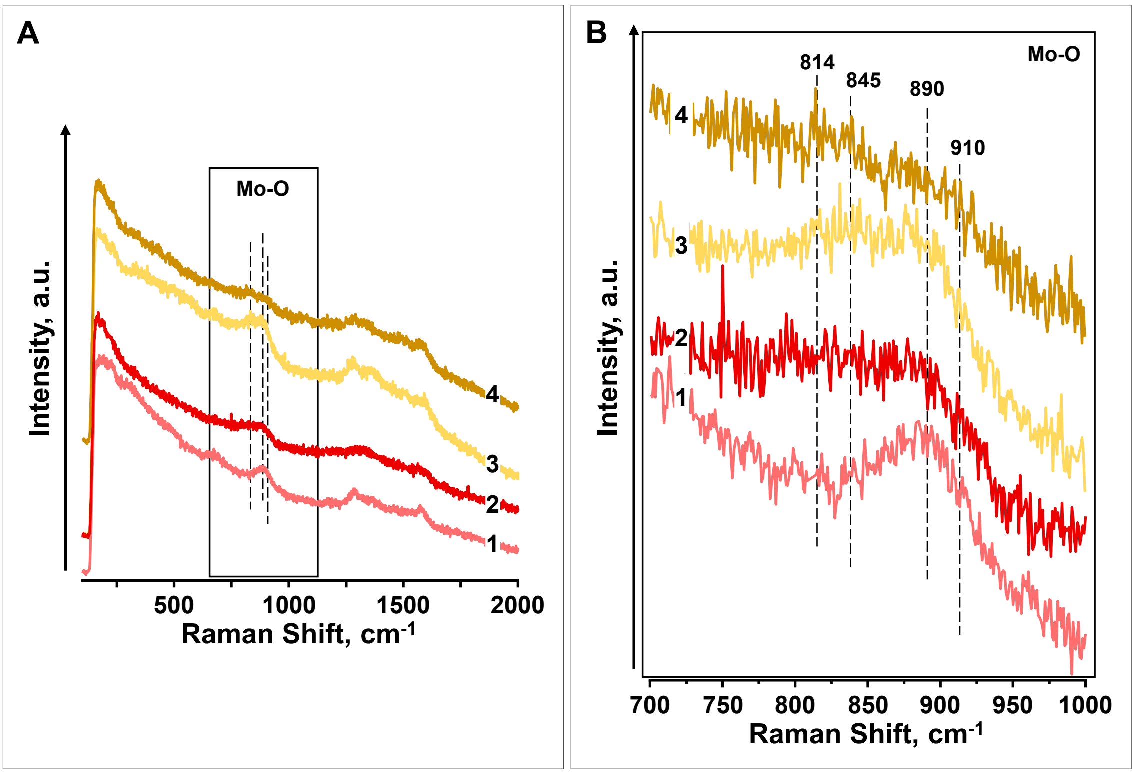

Figure S22. In situ Raman spectroscopic studies on CuZnMo-3.50 catalyst by co-adding ~3.1% H_2O/Ar (recorded with 785 nm diode laser). Conditions: (-1-) H_2 at 200 ºC, (-2-) Ar at 280 ºC, (-3-) ~3.1% H_2O/Ar at room temperature and (-4-) ~3.1% H_2O/Ar at 200 ºC.
